# Microcirculatory Rarefaction in South Asians — A Potential Mechanism for Increased Cardiovascular Risk and Diabetes

**DOI:** 10.1371/journal.pone.0076680

**Published:** 2013-10-07

**Authors:** Alun D. Hughes, Raj Bathula, Chloe Park, Therese Tillin, Nicholas Wit, Simon McG Thom, Nish Chaturvedi

**Affiliations:** International Centre for Circulatory Health, NHLI Division, Faculty of Medicine, Imperial College London and Imperial College Healthcare NHS Trust, London, United Kingdom; National Institutes of Health, United States of America

## Abstract

People of South Asian descent have an increased risk of cardiovascular disease (CVD) and diabetes, but little is known about the microcirculation in South Asian people despite evidence that this plays an important role in the aetiology of CVD. We compared the retinal microcirculation in a population-based sample of 287 middle-aged adults (144 European 143 South Asian) matched for age and sex. Retinal photographs were taken and analysed using a validated semi-automated program and microvascular measures were compared. Blood pressure, anthropometry and fasting bloods were also measured. South Asians had significantly fewer arteriolar and venular vessels and bifurcations. Arterioles and venules were longer and venules were also more tortuous in South Asians. These differences were not explained by adjustment for traditional risk factors including blood pressure, body mass index, diabetes or measures of insulin resistance. People of South Asian descent have rarefaction of the retinal microcirculation compared to age-sex matched individuals of European descent. Reduced microvascular density could contribute to the elevated risk of CVD and impaired glucose tolerance in South Asian people.

## Introduction

People of South Asian descent, whether resident in the Indian subcontinent [1] or other countries, [2] have an increased risk of cardiovascular disease (CVD), particularly myocardial infarction and stoke. Increased levels of diabetes, insulin resistance and hyperglycaemia contribute to this increased risk of CVD, but do not fully explain it. [3].

The microcirculation is increasingly recognized as an important and early determinant of CVD [4]. Diabetes and insulin resistance adversely affect the microcirculation, while conversely microcirculatory dysfunction also contributes to insulin resistance and impaired glucose tolerance. [5] However information on the prevalence of microvascular disease in South Asians and its relationship to CVD and diabetes is limited. It has been suggested that capillary rarefaction could contribute to their risk of CVD; [6] however, there are conflicting reports regarding the prevalence of microalbuminuria in people of South Asian descent with or without diabetes[7]–[10] and comparisons of diabetic retinopathy have been similarly inconsistent.[7], [11]–[13].

Abnormalities of the retinal microvasculature are commonly observed in the general population, and are associated with target organ damage including left ventricular remodelling; [14] increased coronary artery calcification, [15] and impaired myocardial perfusion in individuals without manifest coronary artery disease [16], suggesting a close link between micro- and macro-vascular disease. Retinal microvascular abnormalities also independently predict future diabetes [17] and CVD[18]–[20] suggesting a possible etiological role for microvascular changes.

In view of the high rates of diabetes and CVD in South Asians we investigated whether abnormalities of the retinal microcirculation were more prevalent in this ethnic group, and explored whether any differences observed could be explained by conventional CVD risk factors, including abnormalities of glycaemic control.

## Methods

### Ethics Statement

The study was conducted according to the principles expressed in the Declaration of Helsinki; approved by the Imperial College Healthcare NHS Trust local ethics committee and all participants gave written informed consent.

### Participants

Participants were recruited from the London Life Science Prospective Population Study (LOLIPOP), a multi-ethnic population-based study of chronic disease and its determinants that includes over 25000 individuals identified from 58 primary care lists in West London. [21] Primary care registration in the UK is free and comprehensive, providing a highly representative sampling frame. We identified 300 people at random, aiming for equal numbers of South Asians (all Punjabi Sikh) and Europeans and equivalent numbers of men and women, stratified by 5-year age bands within the range 35–75 years for men and 55–75 years for women. Participants were excluded if they had conditions that precluded retinal photography (e.g. cataract, acute angle closure glaucoma); or prevented full participation in the study. Response rates did not differ significantly by ethnicity (p = 0.2 by Chi^2^ test). Of Europeans and South Asians invited to participate 47% and 48% respectively agreed to participate, 17% and 22% respectively refused to participate and 35% and 30% respectively failed to respond.

### Study Investigations

All participants attended Imperial College Healthcare NHS Trust after an overnight fast having completed a questionnaire detailing demographic data, health behaviours and medical history. Ethnicity was confirmed with the participant, based on self -assessment and place of birth of all 4 grandparents. Height, weight, waist and hip circumference were measured according to a standard protocol. [22] Sitting blood pressure (BP) was measured 3 times according to British Hypertension Society guidelines using a validated automated device (OMRON 705CP) after 5 minutes rest [23] and the average of the final 2 readings was used in analyses. Carotid-femoral pulse wave velocity was measured from the right carotid artery at mid-neck and the common femoral artery at the inguinal ligament using a PulseTrace-PWV device equipped with a 4 MhZ CW directional Doppler probe (Carefusion, Basingstoke, UK) according to recent consensus guidelines. [24] Fasting blood tests were performed for serum glucose (Hexokinase method, Roche, Basel, Switzerland), HbA1c (non-enzymatic method, Toshoh, Tokyo, Japan), lipids (Enzymatic colorimetric method, Roche, Basel, Switzerland) and insulin (ultra-sensitive specific simultaneous ELISA assay using two high affinity monoclonal antibodies, Roche, Indianapolis, USA). Insulin assay cross reactivity with proinsulin was <0.05% and there was no detectable cross reactivity with C-peptide. Fasting serum glucose and insulin were used to calculate HOMA-IR values as a measure of insulin resistance. [25].

### Retinal Photography

Retinal photographs were taken using a Zeiss FF 450 plus fundus camera (Carl Zeiss Ltd.) with a 30° field of view following mydriasis with tropicamide (1%) eye drops and additional phenylephrine (2.5%) eye drops, if necessary. Images were captured on a Basler digital camera and field 1 M of the right eye was subsequently analysed offline as described previously, [19] (field 1 M of the left was used for analysis if the image of the right eye was of inadequate quality). Overall 3% (5/149) of images from either eye from Europeans and 5% (8/151) of images from South Asians were of too poor quality to assess and subsequent analyses refer to the remaining 287 individuals with adequate images; those with un-analysable photographs did not differ significantly from those with adequate images. Retinal measurements were made using a custom written program in Matlab (The Mathworks, Natick, Mass; release 13) with sub-pixel resolution. [26] All vessels up to 3^rd^ order, with diameters ≥10 pixels (approximately 56 µm) were measured. Arteriolar and venular diameters were measured at a series of intensity cross-sections at 2-pixel intervals along its length. At each cross-section, the vessel diameter was measured using a sliding linear regression filter technique and an average calculated for each vessel. [26] Vessel path length was measured along the vessel centre line between bifurcations. To derive a measure of vessel narrowing applicable to both arterioles and venules and unaffected by the refractive properties of the eye, the length:diameter ratio (LDR) was calculated as the ratio of the length of a vessel segment between 2 branching points to its average diameter. Tortuosity was estimated as the difference between the actual path length of the vessel segment (measured by tracking) and the straight line length of the segment divided by the straight line length. Additional measurements included: arteriolar bifurcation optimality ratios (the relationship between arteriolar diameters at branching points) and the arteriolar:venular ratio (AVR). [27] Details of reproducibility of measures have been published elsewhere. [26].

### Statistical Analysis

Population characteristics are presented as means ± SD, or median (25th, 75th percentile) for skewed data. Retinal measures are given as means (95% confidence intervals). Statistical comparisons of unadjusted data were made using a Student’s t-test after log or square root transformation as appropriate. Other comparisons were made following covariate adjustment by multivariate regression analysis with transformation of data as appropriate. P<0.05 was considered statistically significant. All statistical analyses were performed using Stata/IC 11.0 (StataCorp, College Station, Texas, USA).

## Results

Two thirds of participants were male, and the mean age was approximately 62 years (table 1). Height and weight was lower in South Asians but waist hip ratio was higher. There were also fewer current smokers; more people with diabetes and measures of hyperglycaemia, and insulin resistance were more adverse in the South Asian group.

**Table 1:** Characteristics of the two ethnic groups.

	Europeans	South Asians	*p*
N	144	143	
Male gender, n (%)	95 (66)	96 (67)	0.8
Age, y	62.6±6.4	62.1±6.2	0.6
Weight, kg	80.4±17.3	73.8±13.1	<0.001
Height, cm	170±10	166±10	<0.001
BMI, kg/m^2^	27.8±5.2	26.9±4.4	0.2
Waist hip ratio	0.93±0.10	0.97±0.10	0.001
Systolic BP, mmHg	142±18	145±17	0.2
Diastolic BP, mmHg	82±10	82±8	0.5
Heart rate, mmHg	62±10	65±11	0.01
Pulse wave velocity, m/s	9.2 (8.0, 10.3)	9.3 (8.3, 10.6)	0.2
Fasting glucose, mmol/l	5.0 (4.6, 5.4)	5.0 (4.7, 5.9)	0.2
HbA1c, %	5.7 (5.4, 5.9)	6.0 (5.7, 7.0)	<0.001
Fasting insulin, pmol/l	5.4 (3.7, 9.2)	7.6 (4.9, 12.6)	0.004
HOMA-IR	1.2 (0.8, 2.2)	1.8 (1.0, 3.0)	0.004
Diabetes, n (%)	11 (8)	37 (26)	<0.001
Total cholesterol, mmol/l	5.5±1.1	5.2±1.1	0.01
HDL cholesterol, mmol/l	1.4 (1.1, 1.6)	1.3 (1.1, 1.5)	0.04
Fasting triglycerides, mmol/l	1.3 (1.0, 2.5)	1.5 (1.0, 2.0)	0.2
Current smoker, n (%)	75 (52)	4 (3)	<0.001
Hypertensive, n (%)	93 (65)	98 (69)	0.5
Lipid lowering therapy, n (%)	37 (26)	39 (27)	0.8

Values are % (n), mean ± SD, or median (25th, 75th percentile) for skewed data; p values were calculated using the Student’s t-test or the Mann-Whitney U-test for continuous variables and the Chi-squared test for categorical variables. Abbreviations: BMI, body mass index; BP, blood pressure; HbA1c, glycosylated haemoglobin; HDL, high density lipoprotein, HOMA-IR, homeostasis model of the assessment of insulin resistance; WHR, waist hip ratio.

Compared to Europeans, South Asians had significantly fewer arteriolar vessels and bifurcations, and arterioles were longer, and consequently LDR was higher, although arteriolar diameters did not differ significantly (Table 2). The difference in arteriolar number was most evident in 2^nd^ and 3^rd^ order arterioles (Table2).

**Table 2:** Geometrical measures of retinal arterioles by ethnic group.

Measure	Europeans	South Asians	*P*
Arteriolar vessels, n	12.9 [12.2, 13.7]	10.8 [10.2, 11.5]	<0.001
Arteriolar vessels (adjusted)^§^, n	12.8 [12.1, 13.5]	11.0 [10.2, 11.7]	0.001
Arteriolar bifurcations, n	9.9 [9.3, 10.5]	8.4 [7.9, 9.0]	<0.001
Arteriolar bifurcations (adjusted)^§^, n	9.7 [9.2, 10.3]	8.6 [8.0, 9.1]	0.004
1^st^ order arterioles, n	4.4 [4.1,4.7]	3.9 [3.6, 4.2]	0.04
2^nd^ order arterioles, n	5.1 [4.7, 5.4]	4.1 [3.8, 4.4]	<0.001
3^rd^ order arterioles, n	2.1 [1.8, 2.3]	1.4 [1.2, 1.7]	<0.001
Arteriolar diameter, pixels	22.5 [21.9, 23.0]	22.7 [22.2, 23.3]	0.5
1^st^ order arteriolar diameter, pixels	23.0 [22.5, 23.5]	23.3 [22.8, 23.8]	0.5
Arteriolar length, pixels	337 [323, 350]	494 [465, 523]	<0.001
Arteriolar length (adjusted)^§^, pixels	341 [326, 356]	369 [354, 383]	0.01
Arteriolar LDR	15.3 [14.5, 15.9]	16.8 [15.8, 17.7]	0.01
Optimality deviation	0.067 [0.054, 0.080]	0.080 [0.056, 0.104]	0.3
Arteriolar tortuosity (×10^2^)	1.23 [1.01,1.48]	1.46 [1.24, 1.69]	0.2

Values are mean [95% confidence interval] either unadjusted or ^§^ following adjustment for age, sex, systolic blood pressure, heart rate and diabetes. Abbreviations: LDR, length diameter ratio.

South Asians also had fewer venular bifurcations; venules were longer and more tortuous, and venular LDR was higher (Table 3). Since changes in retinal microvascular architecture could result in biased estimates of arteriolar or venular diameter we also compared the diameters of only first order arterioles and venules, but these also did not differ significantly between ethnic groups (Table 2 & 3). There were no other significant differences in retinal vessels between ethnic groups.

**Table 3:** Geometrical measures of retinal venules by ethnic group.

Vessel measure	Europeans (n = 144)	South Asians (n = 143)	*P*
Venular vessels, n	15.1 [14.0, 16.2]	12.0 [11.2, 12.8]	<0.001
Venular vessels (adjusted)^§^, n	14.6 [13.6, 15.6]	12.4 [11.4, 13.4]	0.006
Venular bifurcations, n	12.8 [11.9, 13.7]	10.3 [9.6, 11.0]	<0.001
Venular bifurcations (adjusted)^§^, n	12.9 [12.0, 13.5]	10.5 [9.7, 11.2]	<0.001
Venular diameter, pixels	25.6 [24.9, 26.2]	26.3 [25.5, 27.1]	0.1
1^st^ order venular diameter, pixels	27.0 [26.3, 27.7]	27.8 [27.0, 28.5]	0.1
Venular length*, pixels	257 [244, 269]	282 [267, 299]	0.01
Venular length*(adjusted)^§^, pixels	258 [245, 271]	280 [266, 295]	0.02
Venular LDR	10.6 [10.0, 11.1]	11.7 [10.9, 12.5]	0.02
Venular tortuosity (×10^2^)	0.53 [0.41, 0.67]	0.83 [0.66, 1.02]	0.007
Venular tortuosity (×10^2^) (adjusted)^§^	0.55 [0.42, 0.70]	0.78 [0.62, 0.96]	0.02
AVR	0.87 [0.85, 0.89]	0.85 [0.83, 0.87]	0.1

Values are mean [95% confidence interval] either unadjusted or ^§^ following adjustment for age, sex, body mass index, systolic blood pressure, heart rate, smoking and diabetes. Abbreviations: AVR, arterio-venular ratio; LDR, length diameter ratio.

In all participants there was a positive relationship between systolic blood pressure and arteriolar narrowing, assessed as arteriolar LDR (r = 0.14; p = 0.01); this relationship did not differ by ethnicity (p interaction systolic blood pressure×ethnicity = 0.3). The number of arteriolar bifurcations was also negatively correlated with blood pressure (r = −0.18; p = 0.003) and pulse wave velocity (r = −0.15; p = 0.01). Diabetes was associated with wider arterioles (arteriolar LDR in people without diabetes = 15.7 (15.2, 16.3) pixels, n = 239 vs. 17.5 (15.2, 19.7) pixels in people with diabetes, n = 48; p = 0.03); this difference also did not differ by ethnicity (p interaction diabetes×ethnicity = 0.4). Diabetes was also associated with significantly fewer arteriolar (8.0 (7.2, 8.8), n = 48 vs. 9.4 (8.9, 9.8), n = 239; p = 0.01) and venular (10.2 (8.8, 11.6) ); n = 48 vs. 11.8 (11.2, 12.5), n = 239; p = 0.03) bifurcations.

We proceeded to explore possible explanations for these ethnic differences in the retinal microcirculation through multivariate analysis, using age, sex, blood pressure, heart rate, diabetes (or HbA_1c_, HOMA, or fasting glucose) as covariates for arteriolar measures, and age, sex, blood pressure, BMI, diabetes (or HbA_1c_, HOMA, or fasting glucose) and smoking for venular parameters. These covariates were chosen *a priori* on the basis of previous studies [27], [28]. Adjustment for these covariates alone or in combination only marginally attenuated the ethnic differences and did not abolish statistical significance (Table 2). Further exploratory analyses using variables that differed significantly between ethnic groups (height, weight, waist hip ratio, cholesterol, triglycerides, smoking, use of antihypertensive drugs) also failed to substantially modify the ethnic differences in retinal microvascular measures (data not shown).

## Discussion

South Asians have a reduced number of arterioles and venules and elongated arteriolar and venular segments compared to age-sex matched individuals of European descent. This rarefaction of the retinal microcirculation in South Asians is more prominent in the smaller (higher order) arterioles and is unexplained by blood pressure, diabetes, insulin resistance, or other cardiovascular risk factors known to influence the retinal microvascular architecture.

As far as we are aware no other studies have compared the retinal microcirculation between Europeans and South Asian adults. Owen *et al.* [29] found no difference in tortuosity between European, South Asians and African Caribbean children but did not examine vessel diameter. Jaganathan *et al.* [30] have reported comparisons of central retinal arteriolar and venular equivalents between Malay-, Chinese- and Indian-origin people in Singapore and, as in our study, they found that elevated blood pressure was associated with arteriolar narrowing in all ethnic groups. The authors also reported that retinal arteriolar calibre was wider in people of Malay ethnicity, while Chinese and Indian people did not differ. In contrast, the same group reported that retinal arterioles were narrower in Chinese compared to Malay and Indian children [31] and that diabetes was associated with increased arteriolar diameter irrespective of ethnicity, [32] as we also observed in adults. The number of bifurcations, lengths of vascular segments, or any other measure of rarefaction was not measured in these previous studies.

Others have studied different ethnic groups and found inter-ethnic differences in the retinal microcirculation of adults. In the Multi-ethnic Study of Atherosclerosis (MESA) retinal arteriolar calibre was larger in African Americans and Hispanics than in Whites and Chinese, while venular calibre was largest in African Americans, intermediate in Hispanics and Chinese and smallest in whites. [33] In contrast Tillin *et al.,* [34] reported that people of Black African descent in the UK (African Caribbeans) had narrower arterioles than Europeans. This was explained by their higher blood pressure, but it was also noted that diabetes influenced the relationship between blood pressure and arteriolar diameter. This is consistent with the findings of Mahal *et al.* [35] who reported wider arteriolar calibre in African Caribbean people with diabetes than Europeans with diabetes. Similar observations showing an interaction between arteriolar calibre, diabetes and ethnicity have also been reported in MESA. [33] The current study extends these observations of ethnic differences in the retinal microcirculation by indicating that there appears to be little or no ethnic difference in vascular diameter between South Asians and Europeans, with or without adjustment for blood pressure or diabetes. Furthermore, unlike a previous study comparing African Caribbean people with Europeans we found no difference in optimality deviation, an indicator of microvascular endothelial function. [34] Nevertheless there are significant differences in terms of the architecture of the retinal vasculature, such that South Asians have a sparser vascular tree with longer vascular segments. There was also a significant increase in venular tortuosity; this could be simply attributable to the longer venular segments, although we cannot exclude other mechanisms. While there is evidence of abnormal large artery endothelial function [36] and arterial stiffness [37] in South Asians, current knowledge regarding the microcirculation is limited and what exists is equivocal. Microalbuminuria, an indicator of abnormal renal microvascular function has been reported to be higher [9] or lower [10] in population-based studies of South Asians compared with Europeans, and comparisons of South Asian and European people with diabetes have been equally contradictory. [7], [8] Variable precision of measurement of microalbuminuria; the necessary restriction of retinopathy studies to people with diabetes and, in limitations in sampling frame and/or sample size in some studies may account for these inconsistencies. Previously we have reported that South Asians have impaired cutaneous microvascular post-ischemic hyperaemia compared with Europeans. [38] Nama *et al.* [6] have reported that healthy South Asians have a significantly lower functional and structural cutaneous capillary density compared with healthy Europeans and He *et al*. [39] showed that a modest reduction in salt intake improved capillary rarefaction in White, Black and Asian people with hypertension. Patel *et al.* [40] have also reported abnormalities of retinal arteriolar flicker response in South Asians. It is tempting to speculate that widespread rarefaction and functional impairment of the microcirculation in South Asian could compromise perfusion and flow reserve and contribute to their elevated CVD risk, impaired glucose disposal and susceptibility to diabetes. [41], [42].

Our study has a number of limitations, it is cross sectional and therefore questions of causality cannot be resolved. However it is worth noting that abnormalities in the retinal microcirculation are already apparent in youth and related to low birth weight and post natal growth. [43], [44] The participants in this study were of middle age and above and so we cannot know if the differences observed would be evident in younger individuals. This may be particularly relevant since a proportion of participants are likely to have undiagnosed or subclinical CVD and use of cardiovascular medication was common. The two ethnic groups, as anticipated, differed in several respects, including prevalence of diabetes, glycaemia and smoking habit; while statistical adjustment for these factors did not alter our findings, it is possible that there is residual confounding unaccounted for in our analysis. The strengths of this study are that participants were drawn from a representative population sample and that it employed a well validated technique to examine the retinal microcirculation.

In summary, we have shown rarefaction and elongation of the vascular segments of the retinal microvasculature in people of South Asian descent compared with those of European descent in the UK. Abnormalities of the microvasculature could contribute to the elevated risk of CVD in South Asians.
